# Supplementary material for: CRISPR/Cas9 editing of NKG2A improves the efficacy of primary CD33-directed chimeric antigen receptor natural killer cells
Source: Nat Commun. 2024 Sep 30;15:8439. doi: 10.1038/s41467-024-52388-1 (PMC11442982; doi:10.1038/s41467-024-52388-1)
Supplement: Supplementary file 1 — Supplementary Information [file 41467_2024_52388_MOESM1_ESM.pdf]

Supplementary information for:

CRISPR/Cas9 editing of NKG2A improves the efficacy of primary CD33-directed chimeric antigen receptor natural killer cells

Tobias Bexte[1,2,3,4], Nawid Albinger[1,2,3*], Ahmad Al Ajami[3,5,6], Philipp Wendel[1,2,3,7,8,9], Leon Buchinger[1,2,3], Alec Gessner[3,10], Jamal Alzubi[11,12], Vinzenz Särchen[13], Meike Vogler[13], Hadeer Mohamed Rasheed[14,15], Beate Anahita Jung[14], Sebastian Wolf[3,8,10], Raj Bhayadia[2,3], Thomas Oellerich[3,8,10], Jan-Henning Klusmann[2,3], Olaf Penack[14,16], Nina Möker[17], Toni Cathomen[11,12,18], Michael A. Rieger[3,10,19], Katharina Imkeller[3,5,6], Evelyn Ullrich[1,2,3,8 #]*

1 Goethe University Frankfurt, Department of Pediatrics, Experimental Immunology and Cell Therapy, Frankfurt am Main, Germany

2 Goethe University Frankfurt, Department of Pediatrics, Frankfurt am Main, Germany

3 Goethe University Frankfurt, Frankfurt Cancer Institute, Frankfurt am Main, Germany

4 German Red Cross Blood Service Baden-Württemberg – Hessen, Institute for Transfusion Medicine and Immunohematology, Frankfurt am Main, Germany

5 University Cancer Center (UCT), Frankfurt am Main, Germany

6 Goethe University Frankfurt, University Hospital, Neurological Institute / Edinger Institute, Frankfurt am Main, Germany

7 Institute for Organic Chemistry and Biochemistry, Technical University of Darmstadt, Darmstadt, Germany

8 German Cancer Consortium (DKTK) partner site Frankfurt/Mainz, Frankfurt am Main, Germany

9 German Cancer Research Center (DKFZ), Heidelberg, Germany

10 Goethe University Frankfurt, University Hospital, Department of Medicine II - Hematology and Oncology, Frankfurt am Main, Germany

11 Institute for Transfusion Medicine and Gene Therapy, Medical Center – University of Freiburg, Freiburg, Germany

12 Center for Chronic Immunodeficiency, Faculty of Medicine, University of Freiburg, Freiburg, Germany

13 Goethe University Frankfurt, Institute for Experimental Pediatric Hematology and Oncology, Frankfurt am Main, Germany

14 Charité, University Berlin and Humboldt-University Berlin, Department of Hematology, Oncology and Tumor Immunology, Berlin, Germany

15 Clinical Pathology Department, Faculty of Medicine, Alexandria University, Alexandria, Egypt

16 German Cancer Consortium (DKTK) partner site Berlin, Germany

17 Miltenyi Biotec B.V. & Co. KG, Bergisch Gladbach, Germany

18 German Cancer Consortium (DKTK) partner site Freiburg, Germany

19 Cardio-Pulmonary-Institute, Frankfurt am Main, Germany

* Contributed equally; # Corresponding author. Email: evelyn@ullrichlab.de

Supplementary Figure 1

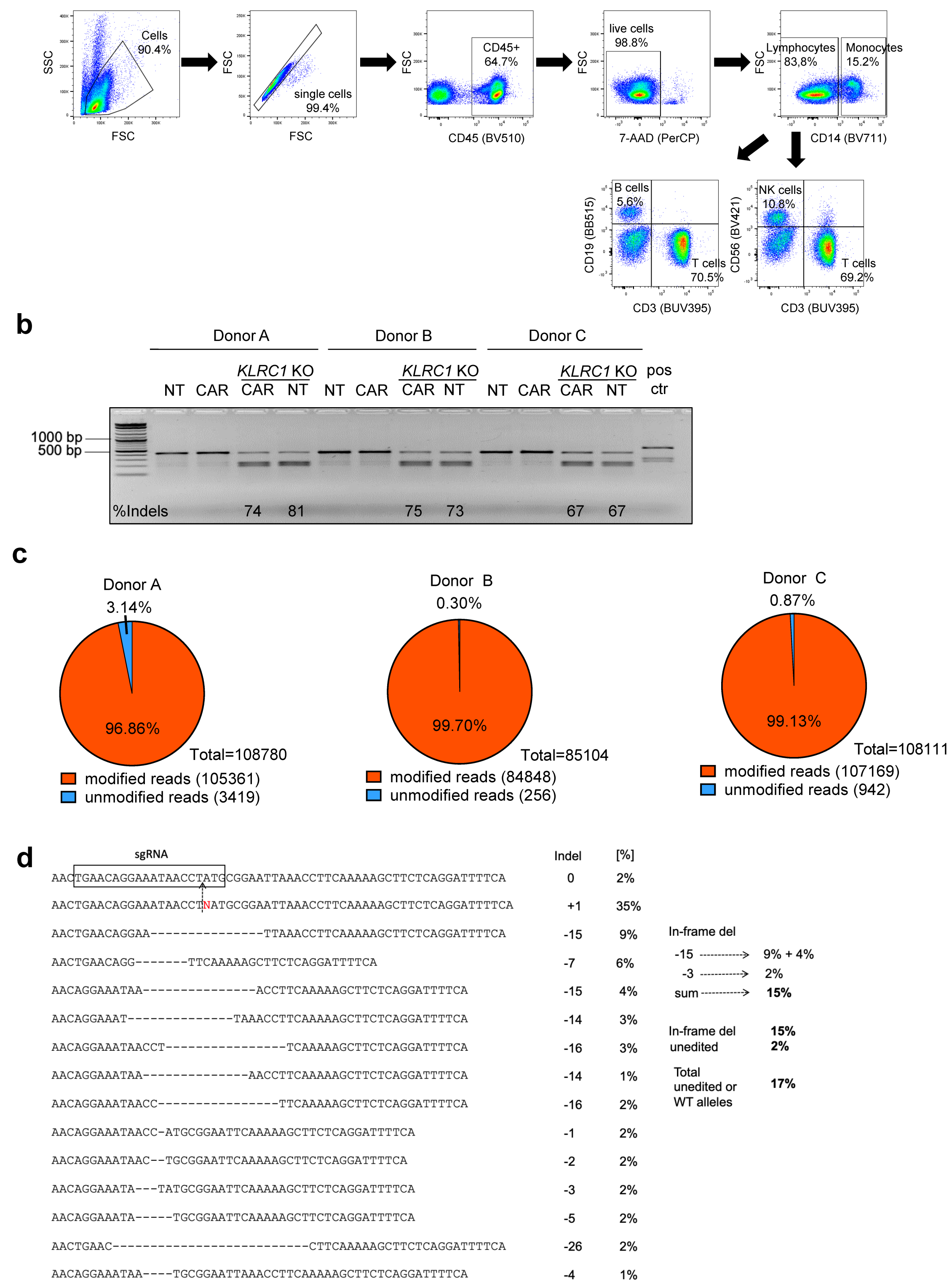

Supplementary Figure 1:

Phenotypic gating strategy of NK cells and genomic analyses of *KLRC1* deletion in NK cells. a: Shown is an exemplary gating strategy of human PBMCs from a healthy donor. Single live cells were determined based on FSC/SSC discrimination and 7-AAD staining. Leucocytes were selected as $CD45^+$ cells and monocytes as $CD45^+CD14^-$ cells. B cells were identified as $CD45^+CD14^-CD3^-CD9^+$, T cells as $CD45^+CD14^-CD3^+$ and NK cells as $CD45^+CD14^-CD3^-CD56^+$. **b:** Frequency of *KLRC1* disruption was evaluated by T7E1 assay in non-transduced (NT), CAR-transduced (+CAR) and without CAR (-CAR) primary NK cells in three different donors (Donor 1, 2, 3). **c**: Frequency of *KLRC1* disruption in CAR33-$KLRC1^{ko}$-NK was evaluated by targeted amplicon next-generation sequencing (NGS) in three different donors. **d**: Window of indels distribution for the ICE using online tool by Synthego.

Supplementary Figure 2

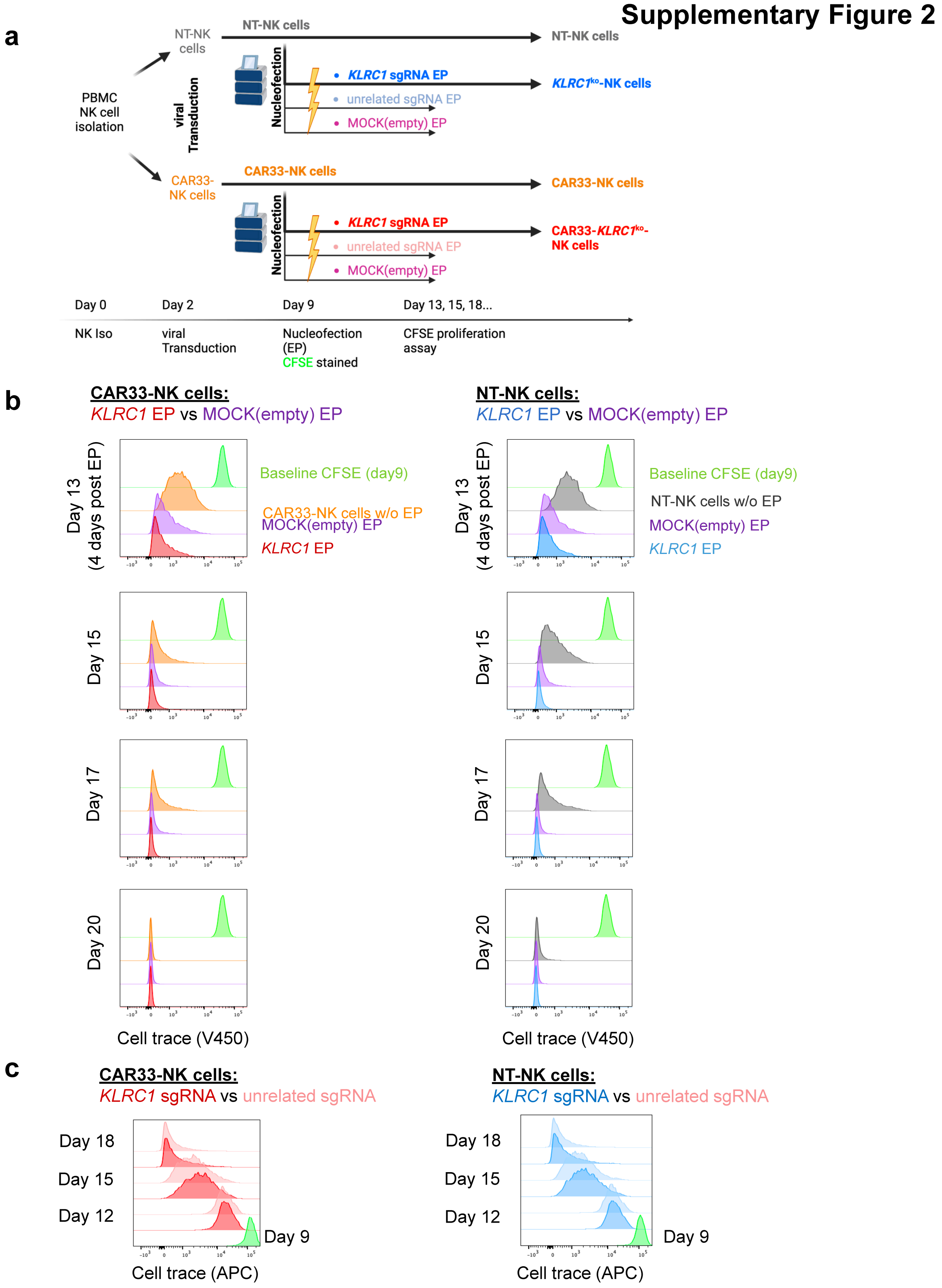

Supplementary Figure 2:

Cell proliferation analyses of nucleofected NK cells. a: Scheme of workflow for nucleofection of NT- and CAR33-NK cells using unrelated sgRNA or empty MOCK nucleofection comparted to sgRNA targeting *KLRC1*. One day prior nucleofection NT- and CAR33-NK cells were labeled with CFSE and flow cytometry measuring was performed after nucleofection of each condition. **b-c**: Flow cytometry analyses of representative FACS plots were shown.

Supplementary Figure 3

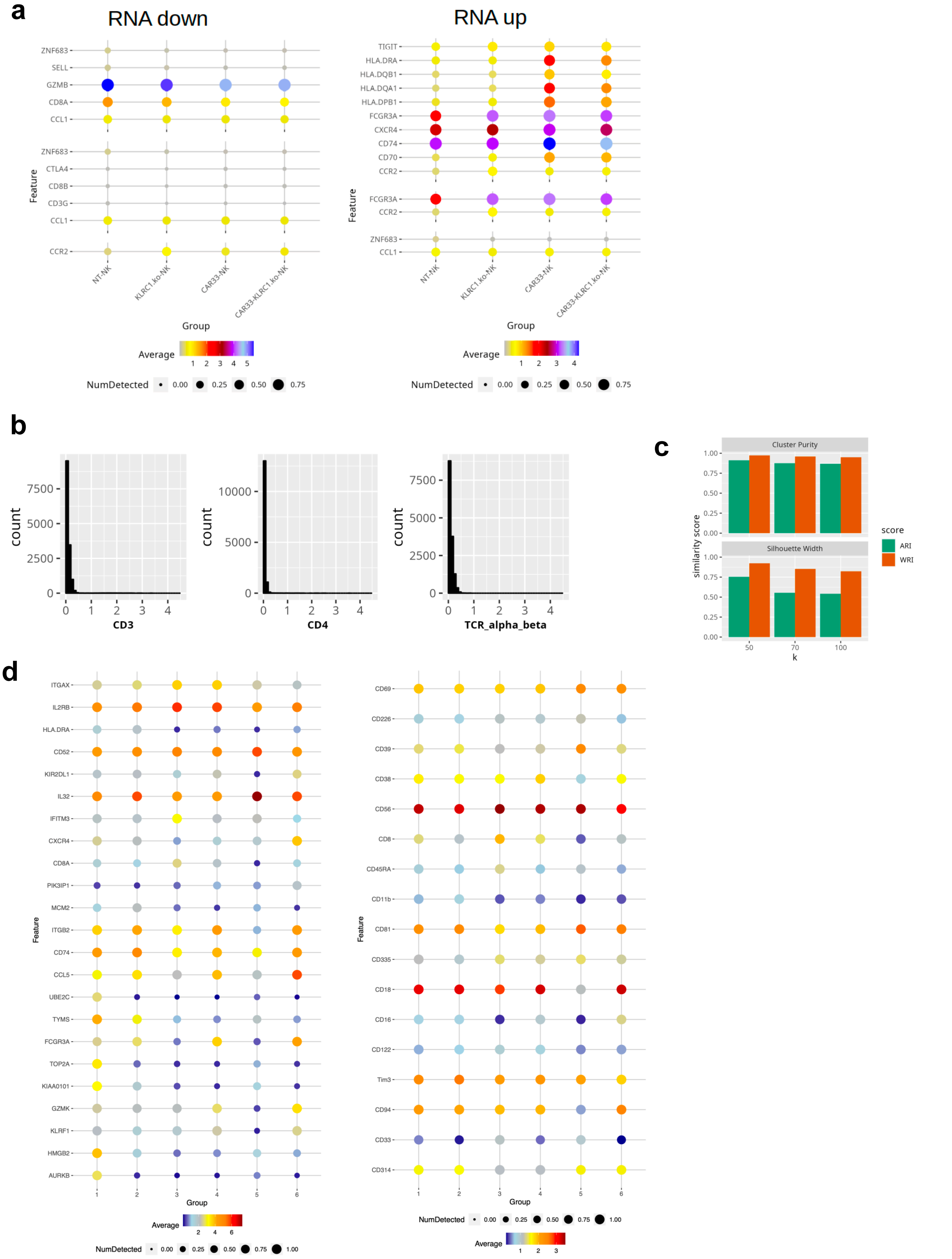

Supplementary Figure 3:

Expression of genes and surface markers of interest across the 6 subclusters. a: Effect of CAR33 (top), *KLRC1*^{ko} (middle), and CAR33-*KLRC1*^{ko} (bottom) on the RNA of donors D1 and D2 NK cells (left graph downregulated RNA; right graph upregulated RNA). The size of the dot reflects the number of cells within a specific condition that express the gene/surface marker. Color codes for average of logarithm-transformed normalized expression values. **b:** Protein expression analyses revealed the absence of T cell receptor expression on the NK cell preparations. **c**: Louvain clustering behavior with three different *k* values was quantified with two metrics: silhouette width and cluster purity. A clustering similarity score between the results of Louvain clustering and the two metrics was calculated in terms of adjusted rand index (ARI) & weighted rand index (WRI). These represent a score between 0 and 1, with 1 indicating a perfect cluster identity. **d**: Average expression of the pathways' features (left graph RNA level; right graph surface marker level). Dot plots show the average expression of the pathways' genes and surface markers across the 6 clusters of donor D1's before co-culture cells. The size of the dot reflects the number of cells within a specific cluster that expresses the gene/surface marker. Color codes for average of logarithm-transformed normalized expression values (RNA) and average of CLR-transformed expression values (surface markers).

Supplementary Figure 4

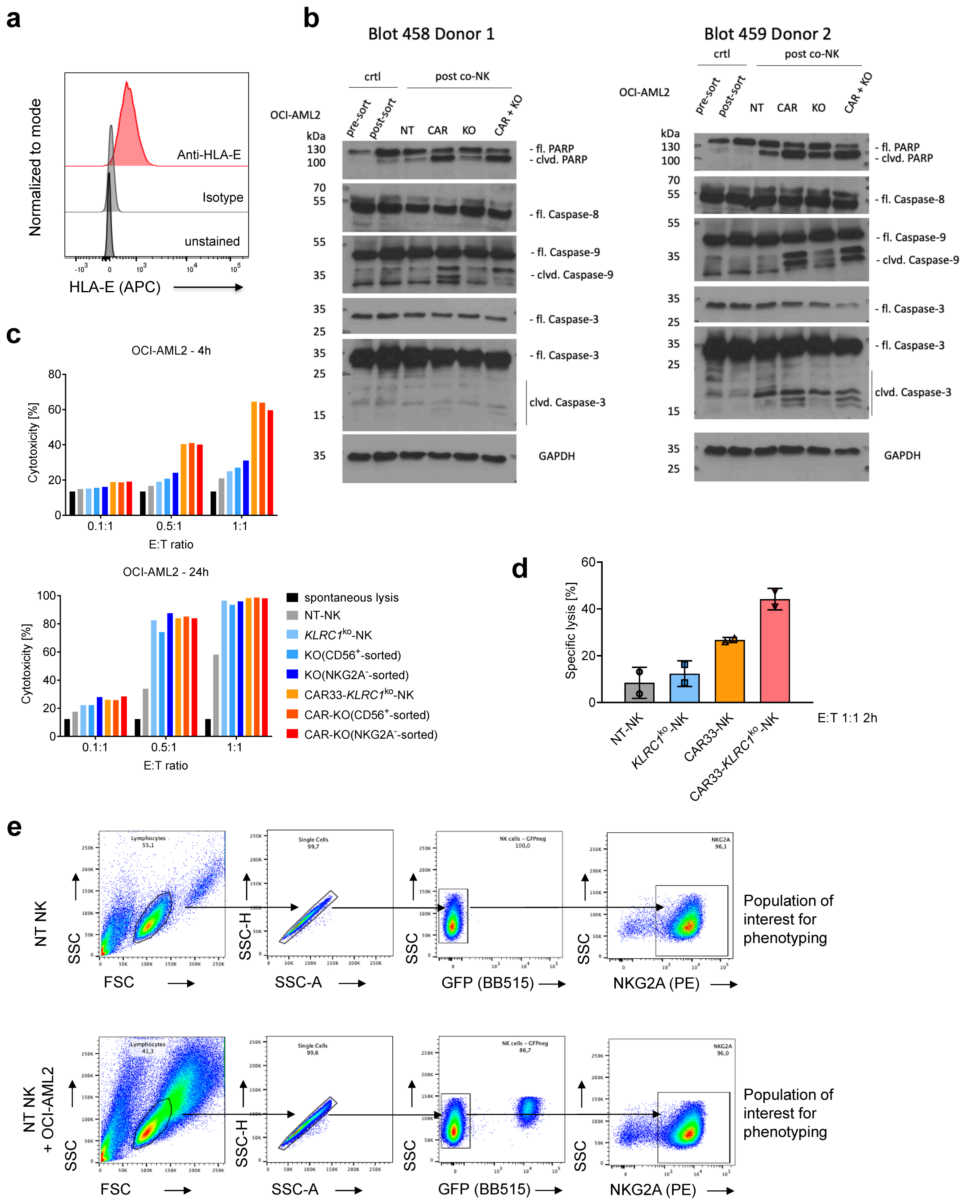

Supplementary Figure 4:

Quality control of anti-AML cytotoxicity of NK cells donors. a: Representative flow cytometry analysis of HLA-E expression on OCI-AML2 cells. **b:** Caspase-cleavage in survived and sorted OCI-AML2 cells following 2-hour NK cell encounter was analyzed using western blot (two representative experiments of three are shown; "clvd" = "cleaved", "ctrl" = "control"). **c:** *KLRC1*^{ko}-NK and CAR33-*KLRC1*^{KO}-NK cells were sorted for NKG2A^{-} cells and co-cultured in a 4h- or 24h-killing assay with OCI-AML2 cells. Control cells were sorted on CD56^{+} cells to exclude sort-dependent effects. Shown is one experiment with one donor (*n*=1). **d:** Flow cytometry-based cytotoxicity assay of thawed NT-NK, *KLRC1*^{ko}-NK, CAR33-NK and CAR33-*KLRC1*^{ko}-NK cells. NK cells deployed in CITE-seq analysis (Donor 1 and Donor 2 2h co-cultivation, *n*=2). **e**: Shown is an exemplary gating strategy of NT NK cells and NT-NK cells with CFSE-labeled/GFP+ OCI-AML2 cells co-incubated for phenotypic analyses. Single live cells were determined based on FSC/SSC and SSC-H/SSC-A discrimination. NK cells were selected as GFP^{-} cells and were gated on phenotypic marker of interested (shown here for NKG2A).

Supplementary Figure 5

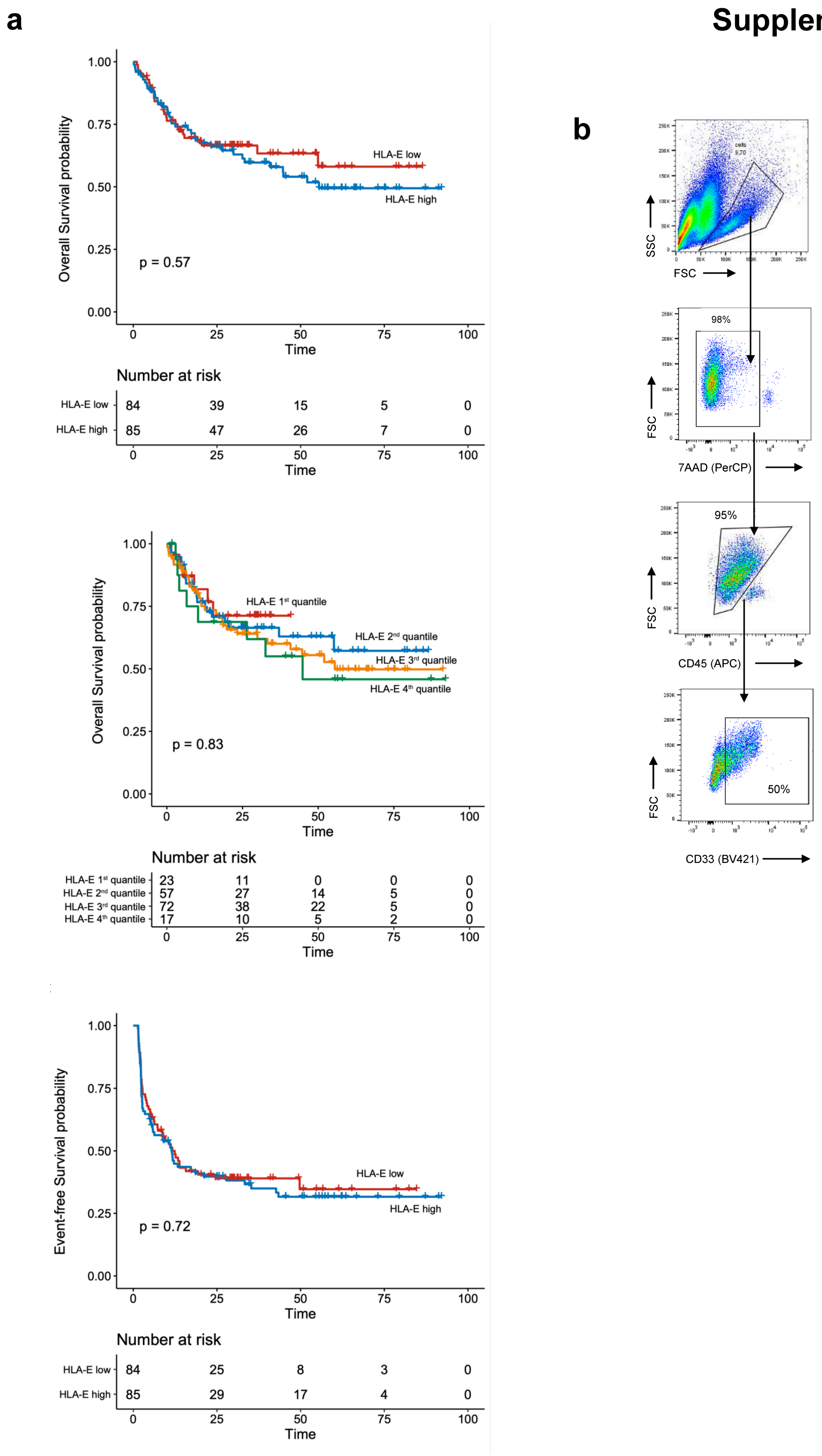

Supplementary Figure 5:

Evaluation of HLA-E expression in clinical AML patient cohorts. a: (top) Kaplan-Meier estimator for overall survival stratified by HLA-E expression above (high) and below (low) median expression, log-rank n-value. **a** (middle): Kaplan Meier estimator for overall survival stratified by HLA-E expression quantiles (1st to 4th), log-rank p-value**. a** (bottom): Kaplan-Meier estimator for event-free survival stratified by HLA-E expression above (high) and below (low) median expression. log-rank o-value. **b:** Gating of patient primary AML-material: Viability, CD33 expression, HLA-E expression and $CD45^{dim}$ AML-blast count of one representative primary BMC sample of AML-patients day one post thawing (one day before co-cultivation with NK cells) were analyzed by flow cytometry.

Supplementary Figure 6

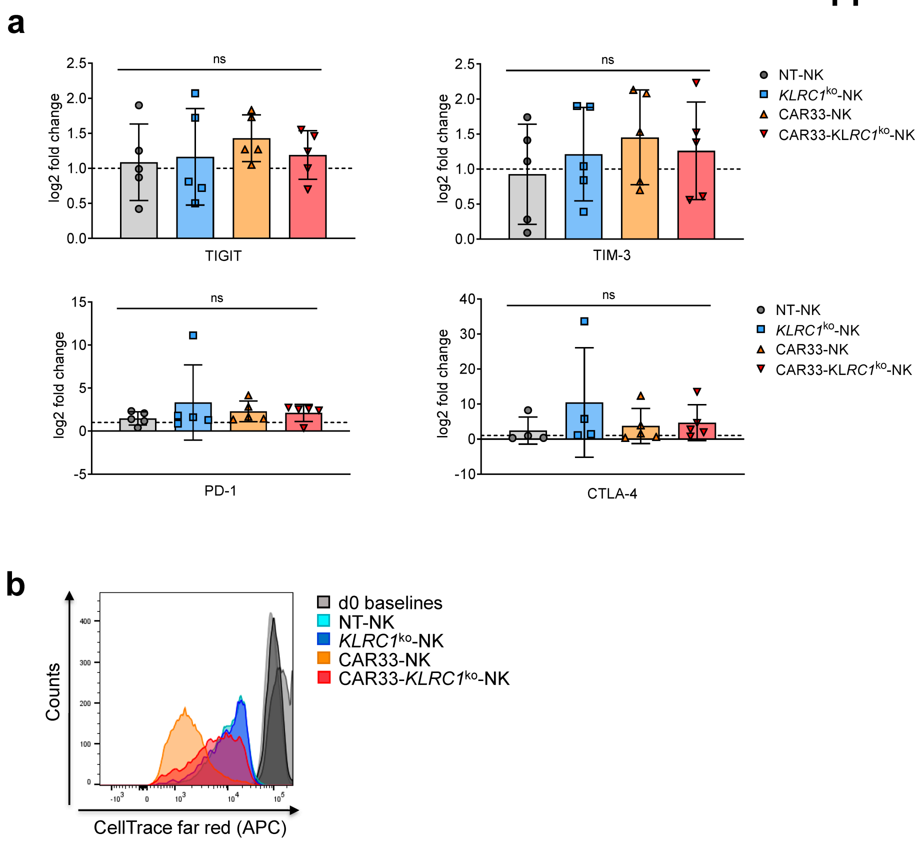

Supplementary Figure 6:

qPCR gene analysis of NK cells. a: qPCR gene expression analysis of NT-, *KLRC1*^{ko}-, CAR33- and CAR33-*KLRC1*^{ko}-NK cells following 2h co-cultivation with OCI-AML2 cells (E:T = 3:1) (n=5). Mean ± SD. Paired Wilcoxon **b:** Proliferation analysis of thawed and CellTrace (far red)-labeled NK cells after co-culture with OCI-AML2 cells for four days (shown is one donor, n=1).

Supplementary Figure 7

a

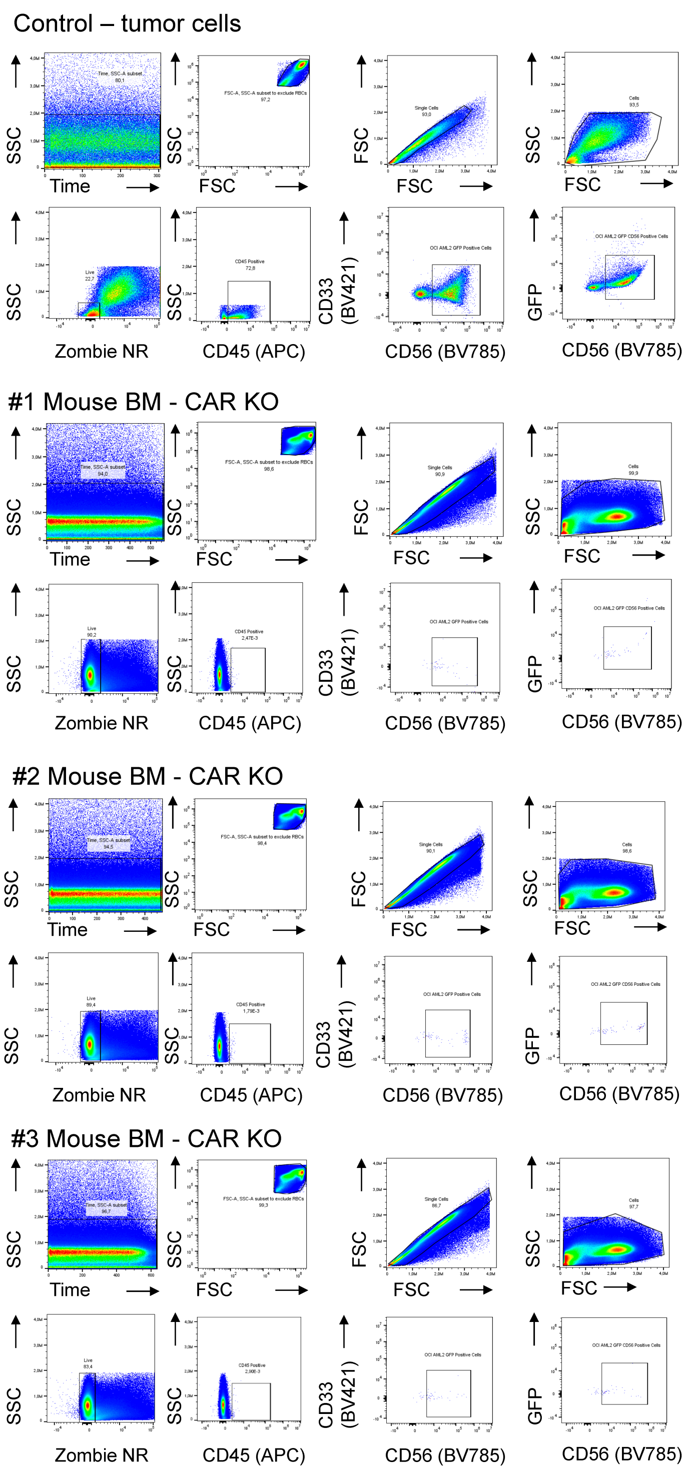

b

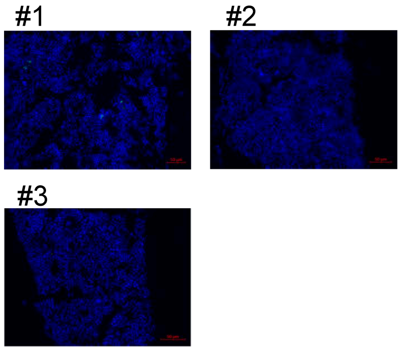

__Supplementary Figure 7:__

Minimal residual disease (MRD) analyses *in vivo*. a-b: MRD analyses using full spectrum flow cytometry: MFC-MRD test positivity is defined as ≥0.1% of CD45-expressing cells with the target immunophenotype (according to The European LeukemiaNet (ELN) MRD Working Party). Human antibodies were used. The panel was first tested on OCI AML2 GFP+ cells to determine the positive markers to use in *in vivo* tracing GFP expression was expressed on 72% of cells and intensity of expression below 104, this may explain the loss of GFP signal. The CD45 negative cells in the MRD analysis plots are mice cells, not human cells (**a**). Confocal microscopy analysis of GFP^{+} AML cells in bone marrow (BM) histology of survival bones (**b**).

Supplementary Figure 8

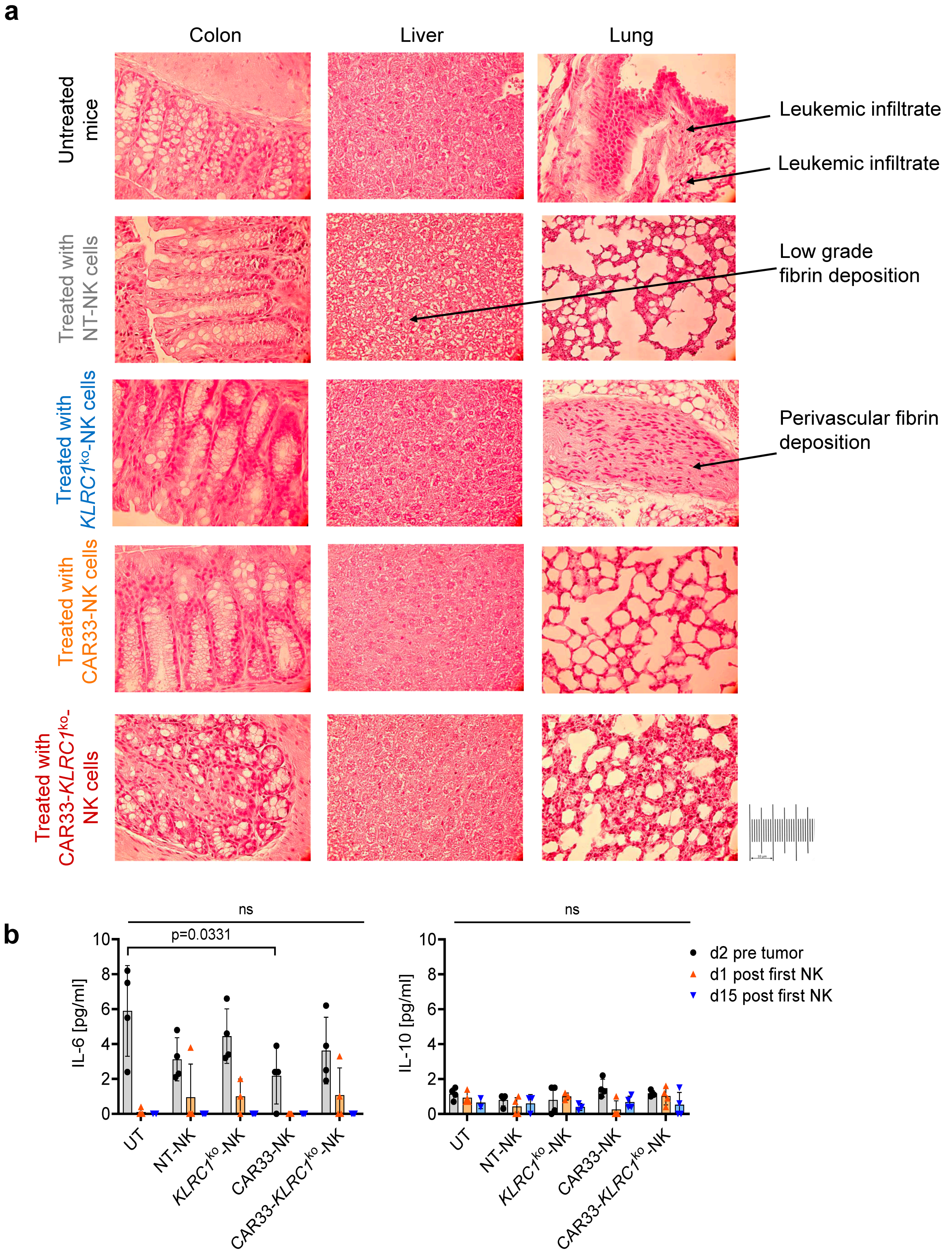

Supplementary Figure 8:

Histology and cytokine reports of *in vivo* experiments. a: Histology report of colon, liver and lunge organs of mice treated with different NK cells or left untreated. **b**: Cytokine levels in blood from the different NK cell-treated NSG-SGM3 mice were shown at different time points (BD Cytometric Bead Array (CBA; BD Biosciences)). Mixed effects analysis (Multiple comparison).

Supplementary Table 1: Clinical characteristics of primary AML patients used for cytotoxic analyses *in vitro*.

Sample	Sex	Age group	Precursor	WHO 2016	Karyotype	Complex_Karyotype	NPM1	CBFB::MHY11	FLT3	FLT3.ITD.Ratio	CEBPa_biallelic	MLL.Translocation	t.9.11...MLL.MLLT3	t.6.9.	inv.3.	RUNX1.RUNX1T1	BCR.ABL	RUNX1	ASXL	TP53	del.5.	Mutations (NGS)	Additional mutations	ELN2017	FAB	High-risk molecular subset
**1**	w	<50	de novo	AML with mutated NPM1	46, XX	no	mut	no	ITD und TKD	0.06	no	no	no	no	no	no	no	no	no	no	no	DNMT3A, IDH1, NPM1, KMT2C, PMS2, PTEN, SETBP1		favourable	M4	
**2**	w	>65	de novo	AML with mutated NPM1	46,XX	no	mut	no	TKD		no	no	no	no	no	no	no	no	no	no	no			favourable	M4	
**3**	w	50-65	de novo	AML with mutated NPM1	46, XX	no	mut	no	ITD	0.784	no	no	no	no	no	no	no	no	no	no	no	ETV6, NPM1, FLT3-ITD		intermediate	M4	
**4**	w	50-65	de novo	AML with mutated NPM1	46, XX	no	mut	no	ITD	0.8	no	no	no	no	no	no	no	no	no	no	no	FLT3, NPM1, DNMT3A		Intermediate	M4	
**5**	w	<50	tAML	AML with MLLT3::KMT2A	46, XX, t(9;11)(p22;q23)[13], 46, XX [7]	no	WT	no	WT		no	no	yes	no	no	no	no	no	no	no	no	IDH1		intermediate	M1	rather yes
**6**	w	<50	de novo	AML with mutated RUNX1	46,XX	no	WT	no	TKD		no	no	no	no	no	no	no	yes	no	no	no	DNMT3A, SF31A, SETBP1, PTEN		adverse	M2	yes

**7**	m	<50	de novo	AML	46, X, -Y, der(1)t(1;3)(p12;p25), der(3)t(1;3)(p21;q13)t(3;9)(p25;q33), der(8)t(8;14)(p21;q23), der(9)t(3;9)(q13;q33), der(14)t(14;22)(q22;q13), t(18;22)(q21;p13), +20, +21, der(21)(22qter->22q12::21p13->21p11::22q12->22q11::21p11->21qter)x2, -22 [10]<br><br>46, XY [10]	yes	WT	no	ITD	0.49	no	no	no	no	no	no	no	no	no	no	no			adverse	M4	yes
**8**	m	>65	de novo, AML-MR nach WHO2022	AML with mutated RUNX1 (WHO2016), AML-MR (WHO2022)	46, XY [20]	no	WT	no	TKD	0	CEBPa (benigne)	no	no	no	no	no	no	yes	yes	no	no		NF1, NRAS, PRPF8, PTPN11, STAG2, TET2, SRSF2	adverse		yes
**9**	w	50-56	de novo, AML-MR nach WHO2022	AML with GATA2::MECOM	45, X, dic (X,9)(q11;q11), inv(3)(q21;q26), den(7)t(X;7)(q21;p21)t(X;17)(q?26;q11),del(7)(q211q36), der(17)t(9	yes	WT	no	ITD	0.17	no	no	no	no	yes	no	no	no	no	no	no			adverse		yes

					;17)(q22; q11) [20]																					
**10**	m	50-56	de novo, AML-MR nach WHO2022	AML with myelodysplasia-related changes (WHO2016), AML-MR (WHO2022)	46, XY, del(11)(q13q24) [2]<br>45, XY, -7, del(11)(q13q24) [17]<br>46, XY [3]	no	WT	no	no		no	no	no	no	no	no	no	no	no	no	no		NF1, SETBP1, CBL, -7,- 11	adverse		yes

Further characteristic of the cohort: https://www.cell.com/cms/10.1016/j.ccell.2022.02.006/attachment/51334da3-3ef0-4896-9b6a-495e37174ace/mmc7.zip

Supplementary Table 2: Antibody list.

Antibody	Supplier Name	Catalog Number	Clone Name	Lot Number	Dilution
Brilliant Violet 421™ Mouse anti-human CD33 Antibody	Biolegend, San Diego, California, USA	303415	WM 53	B388992	1:500
Spark Violet™ 423 Mouse anti-human CD19 Antibody	Biolegend, San Diego, California, USA	302281	HIB19	B356406	1:500
Brilliant Violet 605™ Mouse anti-human CLL-1 [CLEC12A] BV605	BD biosciences, California, USA	742931	50C1	3201430	1:300
Brilliant Violet 750™ Mouse Anti-Human CD34	BD biosciences, California, USA	747036	563	3235281	1:1000
Brilliant Violet 786™ Mouse Anti-Human CD56	BD biosciences, California, USA	564058	NCAM16.2	3192744	1:300
PE Mouse anti-human CD133 Antibody	Biolegend, San Diego, California, USA	397903	W6B3C1	B365309	1:200
PE/Dazzle™ 594 Mouse anti-human CD7 Antibody	Biolegend, San Diego, California, USA	343119	CD7-6B7	B376861	1:200
PerCP/Cyanine5.5 Mouse anti-human CD3 Antibody	Biolegend, San Diego, California, USA	317336	OKT3	B227415	1:200
PE/Cyanine7 Mouse anti-human CD117 (c-kit) Antibody	Biolegend, San Diego, California, USA	313211	10D2	B384495	1:500
APC Mouse anti-human CD38 Antibody	Biolegend, San Diego, California, USA	303509	HIT2	B320769	1:200
Alexa Fluor® 647 Mouse Anti-Human CD123	BD biosciences, California, USA	563599	9F5	2353937	1:300
Alexa Fluor® 700 Mouse anti-human CD14 Antibody	Biolegend, San Diego, California, USA	367113	63D3	B396761	1:300
APC/Cyanine7 Mouse anti-human HLA-DR Antibody	Biolegend, San Diego, California, USA	307617	L243	B353658	1:500
APC/Fire™ 810 Mouse anti-human CD45 Antibody	Biolegend, San Diego, California, USA	304075	HI30	B374267	1:500

Flow cytometry antibodies (in vitro):

Antibody	Supplier Name	Catalog Number	Clone Name	Lot Number	Dilution
CD159a-PE (NKG2A) Mouse-anti human	Beckman Coulter, USA	IM3291U	Z199	44	1:20
BD Horizon™ BUV395 Mouse Anti-Human CD3	BD Horizon™, USA	564001	SK7 (Leu-4)	9322768	1:20
BD Horizon™ BV786 Mouse Anti-Human CD56	BD Horizon™, USA	564058	NCAM16.2	3292030	1:20
BD™ CD45 APC	BD Biosciences, California, USA	340910	2D1	3266906	1:20
CD33 Antibody, anti-human, REAfinity™	Miltenyi Biotec, Bergisch Gladbach, Germany	130-111-136	REA775	5190917134	1:50
Biotin Antibody, REAfinity™	Miltenyi Biotec, Bergisch Gladbach, Germany	130-110-951	REA746	5240506611	1:50
CD33 CAR Detection Reagent, human	Miltenyi Biotec, Bergisch Gladbach, Germany	130-127-642	REA746	5240506611	1:50
BD Pharmingen™ PE Mouse Anti-Human CD33	BD Pharmingen™, USA	561816	WM53	7068753	1:20
BD Horizon™ BV421 Mouse Anti-Human CD33	BD Horizon™, USA	562854	WM53	3229681	1:20
APC anti-human HLA-E Antibody	Biolegend, San Diego, California, USA	342606	3D12	B367827	1:20
BD Horizon™ BV510 Mouse Anti-Human CD45	BD Horizon™, USA	563204	Hi30	9344066	1:20
Brilliant Violet 711™ anti-human CD14 Antibody	Biolegend, San Diego, California, USA	301838	M5E2	B281243	1:20
BD Horizon™ BB515 Mouse Anti-Human CD19	BD Horizon™, USA	564456	HIB19	9129583	1:20
BD Horizon™ BUV395 Mouse Anti-Human CD3	BD Horizon™, USA	564001	SK7(Leu-4)	9322768	1:20
BD Horizon™ BV421 Mouse Anti-Human CD56	BD Horizon™, USA	562751	NCAM16.2	9171804	1:20
BD Horizon™ PE-CF594 Mouse Anti-Human CD16	BD Horizon™, USA	562293	3G8	9186082	1:20
BD Horizon™ BV605 Mouse Anti-Human CD69	BD Horizon™, USA	562989	FN50	7145905	1:20

BD OptiBuild™ BV421 Mouse Anti-Human CD226 (DNAM1)	BD Biosciences, California, USA	742493	DX11	9099726	1:50
CD314-PE (NKG2D)	Beckman Coulter, USA	A08934	ON72	200047	1:50
CD279 (PD1) Antibody, anti-human	Miltenyi Biotec, Bergisch Gladbach, Germany	130-096-164	PD1.3.1.3	5170313174	1:50
BD OptiBuild™ BV605 Rat Anti-Human CD366 (TIM-3)	BD OptiBuild™, USA	747961	344823	2322182	1:20
BD OptiBuild™ BUV395 Mouse Anti-Human TIGIT	BD OptiBuild™, USA	747845	741182	3346633	1:20
CD94 Antibody, anti-human, REAfinity™	Miltenyi Biotec, Bergisch Gladbach, Germany	130-123-678	REA113		1:50
BD Horizon™ BV421 Mouse Anti-Human CD57	BD Horizon™, USA	563896	NK-1	0142919	1:20
BD Horizon™ BV421 Mouse Anti-Human CD335 (NKp46)	BD Biosciences, California, USA	564065	9E2	7195560	1:50
BD OptiBuild™ BV711 Mouse Anti-Human CD336 (NKp44)	BD Biosciences, California, USA	744303	p44-8	8113717	1:50
CD337 (NKp30) Antibody, anti-human	Miltenyi Biotec, Bergisch Gladbach, Germany	130-092-483	AF29-4D12	5201101248	1:50

Immunofluorescence Antibodies:

Antibody	Supplier Name	Catalog Number	Lot Number	Dilution
Chicken Anti-Green Fluorescent Protein (GFP) Antibody	Aves Labs	GFP-1010	GFP917979	1:1000
Goat anti-Chicken IgY (H+L) Secondary Antibody, Alexa Fluor™ 488	Invitrogen, USA	A11039	2566343	1:1000
BD Pharmingen™ 7-AAD	BD Biosciences, California, USA	559925	2164283	1:200
Annexin V Red Dye	Sartorius, USA	4641		1:200
CellTrace™ CFSE Cell Proliferation Kit	Invitrogen, USA	C34554	2668608	1:1000

Western plot Antibodies:

Antibody	Supplier Name	Catalog Number	Source	Dilution
Caspase-3	Cell Signaling, Beverly, MA, USA	9662S	rabbit	1:1000
Caspase-8	Enzo, Lörrach, Germany	ADI-AAM-118-E	mouse	1:1000
Caspase-9	Cell Signaling, Beverly, MA, USA	9502S	rabbit	1:1000
GAPDH	BioTrend, Cologne, Germany	NB-29-00852	mouse	1:5000
PARP	Cell Signaling, Beverly, MA, USA	9542S	mouse	1:1000

Supplementary Table 3: Primers used for qRT-PCR.

G6PD (fwd: 5’-ATCGACCACTACCTGGGCAA-3’, rev: 5’-TTCTGCATCACGTCCCGGA-3’), RPII (fwd: 5’-GCACCACGTCCAATGACAT-3’, rev: 5’-GTGCGGCTGCTTCCATAA-3’), 18S-rRNA (fwd: 5’-CGCAAATTACCCACTCCCG-3’, rev: 5’-TTCCAATTACAGGGCCTCGAA-3’), TRAIL (fwd: 5’-CCTAGAGAGTAGCAGCTCACA-3’, rev: 5’-CAGAGCCTTTTCATTCTTGGA-3’), FasL (fwd: 5’-CATTTAACAGGCAAGTCCAACTCAAGGTCCA-3’, rev: 5’-AGTTCCTCATGTAGACCTTGTGGCTCAG-3’), Perforin (fwd: 5’-TAGAAGTGATGTGAGTGGTGGC-3’, rev: 5’-CATGGAGCTGGAATCCCGTATAGAG-3’), Granzyme B (fwd: 5’-GATGCAGGGGAGATCATCGG-3’, rev: 5’-CTCGTATCAGGAAGCCACCG-3’), TIM-3 (fwd: 5’-ACTTACGGGACTCTGGAGCA-3’, rev: 5’-GGCCAAAGAGATGAGGCTTA-3’), NKG2D (fwd: 5’-CTGTAGCCATGGGAATCCGT-3’, rev: 5’-GCCACAGTAACTTTCGGTCAA-3’)

Supplementary Table 4: BD AbSeq oligonucleotide-conjugated antibodies.

Antigen	Clone	Sequence	Cat#
CD3	SK7	AAAGGTAGAGTGTATTGACGTCGGTGTAGGTTGATT	940000
CD4	SK3	TCGGTGTTATGAGTAGGTCGTCGTGCGGTTTGATGT	940001
CD19	SJ25C1	TAGTAATGTGTTCGTAGCCGGTAATAATCTTCGTGG	940004
CD14	MφP9	TGGCCCGTGGTAGCGCAATGTGAGATCGTAATAAGT	940005
CD56	NCAM16.2	AGAGGTTGAGTCGTAATAATAATCGGAAGGCGTTGG	940007
CD25	2A3	AGTTGTATGGGTTAGCCGAGAGTAGTGCGTATGATT	940009
CD45RA	HI100	AAGCGATTGCGAAGGGTTAGTCAGTACGTTATGTTG	940011
CD127	HIL-7R-M21	AGTTATTAGGCTCGTAGGTATGTTTAGGTTATCGCG	940012
CD38	HIT2	GTCAACGATGGGTAGCGGTAGAAATAACGGAACTGG	940013
CD279	EH12.1	ATGGTAGTATCACGACGTAGTAGGGTAATTGGCAGT	940015
CD28	CD28.2	TTGGTTTCGTAAGCGGCTAAGCGTATCTCGTGTTTG	940017
CD69	FN50	CAATAACGGGTCATAGTAAGTCGCGAGTAAGAGGGC	940019
CD34	581	TGGGTGTATTACGGTTAGTTTATGCGCGAAGGTGTT	940021
CD45RO	UCHL1	TGAGAGGTTATTGGGCGTATGACTTCGGTGATTGTG	940022
IgD	IA6-2	TGAGGGATGTATAGCGAGAATTGCGACCGTAGACTT	940026
IgG	G18-145	AGGTAGGTTATCGTAGGGTAGACTTAGCGGGCATTG	940027
CD7	M-T701	GTATGTAGGTCTTATGTGTTGGCGTAGTATGCGTTT	940029
CD33	WM53	GTGTTAGTGATTTGATAGGACGCGTTACGAGAGATT	940031
CD152	BNI3	TAGTATCCGTAGTAGTTATCTGCCCGTTCGTTATGC	940034
CD95	DX2	GGCCCGTTAGAGTTGGTATCCGTATGAAGGTTAGCT	940037
CD5	UCHT2	ACGAAGCGAGCGAAGAACCTATGCGATTGAGTAAGT	940038
CD2	RPA-2.10	AAACGTAGATTAGAGCCGGGTATGTCGCAACTGATT	940046
CD21	B-ly4	GTATTCGCGTATTGTCAGTCGGTAGGGTTATGGTCT	940048
CD40	5c3	GGTGTAATTGGGCTAGAACGTATATGCGGTAAGGCG	940049
CD81	JS-81	TTAGATTGACGGTCGAAGAGTTACGCCTGATTGTGT	940052
CD314	1D11	TTGAAATGCGATGAGACGTAGAGCGATGTAGGTAGC	940061
CD335	9E2/NKp46	CAATTTGTTCGCGTTTAGTAGTCGTCGTCTTATGGG	940064
CD366	D3	TAGGTAGTAGTCCCGTATATCCGATCCGTGTTGTTT	940066
CD103	Ber-ACT8	AAATAGTATCGAGCGTAGTTAAGTTGCGTAGCCGTT	940067
CD161	DX12	GTTATGGTTGTCGGTAGAGTATCGTGTTGCGTTAGT	940070
CD39	TU66	TGTTGTCTTGTACGGCTTGAGTCGGGTTAATTCGGG	940073
TCRαβ	IP26	TTGCGTCGGATTATTAGTTCGGGTATTATGCGGTGC	940074
CD226	DX11	GAGTTTATGATTCGTTTCTTCGGTAGTTCGTCGCTT	940075
CD94	HP-3D9	GAGGTTAGGATAGGTGTACGGGTCGAGTTGAATTCT	940081
CD18	6.7	AGGCGTTCTAAATTGTCGTATGGTGCGGGTATGTCT	940086
CD26	M-A261	TGTAGGTTGCGCGGTTATTAGGGTATTATCGATCTG	940101
CX3CR1	2A9-1	GGGTTCACGAGGTTTAAAGCGGTAGTATAGGATGCC	940216
CD122	Mik-β3/IL2RB	TTAAAGAGATTCGTGGGTATTGGCGCAGTCATTCCT	940232
CD102	CBR-IC2/2	GTTGGATTGGGTCGGTAGGATTTGGTCGGGTTTAGT	940241
CD11b	ICRF44	ATGGATTCGGTTCGGTGTTTGGATAGATAGGCTGCG	940266
CD22	HIB22	TGGTTCGTGACTGTATAGGCTTAGCTTAGGCAATTT	940273
IgM	G20-127	TTTGGAGGGTAGCTAGTTGCAGTTCGTGGTCGTTTC	940276
CD215	JM7A4	GGTACTGATGTGGCGATATATAGACGAAACTGGGTG	940290
CD1d	CD1d42	GTTAGGATTATTGACGTACCGAGTTAGGAGTGATTG	940296
CD8	SK1	AGGACATAGAGTAGGACGAGGTAGGCTTAAATTGCT	940305
CD16	B73.1	GCGTTTGTAGTAAGGAGATCTGCGAATAGCGTAGGG	940314

CD27	L128	CTGTTATTATAGCGAGCGTTGATTTCCGGGTTAGGT	940319
TCRγδ	11F2	CTCGTGGGTTAGGCTTGATCGTAGTTATGTATGGTT	940365
CD197	2-L1-A	AATGTGTGATCGGCAAAGGGTTCTCGGGTTAATATG	940394

Supplementary Table 5: Genes of Human Immune Response Targeted Panel.

ADA	CCR3	CD163	DUSP1	HAVCR2	IL6	LAMP1	PRDM1	TLR8
ADGRE1	CCR4	CD200	DUSP2	HLA-A	IL6R	LAMP3	PRDM1	TLR9
ADGRG3	CCR5	CD209	DUSP4	HLA-C	IL7R	LAP3	PRF1	TMEM97
AIM2	CCR6	CD244	EBF1	HLA-DMA	IL9	LAT	PSEN1	TNF
ALAS2	CCR6	CD247	EGR1	HLA-DMB	IL9R	LAT2	PTGDR2	TNFRSF1B
ANXA5	CCR7	CD274	EGR3	HLA-DPA1	IL12A	LCK	PTPRC	TNFRSF4
AOC3	CCR8	CD300A	ELANE	HLA-DPB1	IL12RB1	LEF1	PTTG2	TNFRSF8
APOBEC3G	CCR9	CEACAM8	ENTPD1	HLA-DQA1	IL12RB2	LGALS1	PYCR1	TNFRSF9
APOE	CCR10	CHI3L1	EOMES	HLA-DQB1	IL13	LGALS3	QPCT	TNFRSF13C
AQP9	CD1A	CHI3L2	EPX	HLA-DRA	IL15	LGALS9	RGS1	TNFRSF17
ARG1	CD1B	CLC	F5	HLA-DRB3	IL15RA	LIF	RNASE2	TNFRSF18
ARL4C	CD1C	CLEC2D	F13A1	HMGB2	IL17A	LILRB4	RNASE6	TNFRSF25
ATF6B	CD2	CLEC4D	FAM65B	HMMR	IL17F	LIPA	RORA	TNFSF8
AURKB	CD2	CLEC4E	FAM129C	ICAM1	IL18	LRRC32	RORC	TNFSF10
AZU1	CD3D	CLEC10A	FAS	ICOS	IL18R1	LTA	RPN2	TNFSF13
B3GAT1	CD3E	CMKLR1	FASLG	IER3	IL18RAP	LTB	RUNX3	TNFSF13B
BACH2	CD3G	CMTM2	FBXO22	IER5	IL21	LY86	S1PR1	TNFSF14
BAX	CD4	CNOT2	FCER1A	IFITM2	IL22	LYN	S100A9	TOP2A
BCL2	CD5	CNTNAP3	FCER1G	IFITM3	IL23R	MCM2	S100A10	TPSAB1
BCL2A1	CD6	CPA3	FCER2	IFNA1	IL25	MCM4	S100A12	TRAC
BCL6	CD7	CR2	FCGR3A	IFNG	IL31	MGST1	SELL	TRAT1
BCL11B	CD8A	CSF2	FCN1	IFNGR1	IL32	MITF	SELPLG	TRBC2
BIN2	CD8B	CSF3	FLT3	IGBP1	IL33	MKI67	SEMA7A	TRDC
BIRC3	CD9	CST7	FN1	IGHA1_secreted	IRF4	MME	SLAMF1	TREM1
BLK	CD14	CTLA4	FOSB	IGHD_membrane	IRF8	MMP9	SLC7A7	TRIB2
BLNK	CD22	CTSD	FOSL1	IGHE_secreted	ITGA4	MMP12	SLC25A37	TSPAN32
BPI	CD24	CTSG	FOXO1	IGHG1_membrane	ITGAE	MS4A1	SNCA	TXK
BTG1	CD27	CTSW	FOXO3	IGHG1_secreted	ITGAL	MYC	SPOCK2	TYMS
BTLA	CD28	CX3CR1	FOXP1	IGHG2_secreted	ITGAM	MZB1	SPP1	UBE2C
C1QA	CD33	CXCL1	FOXP3	IGHG3_secreted	ITGAX	NAMPT	STAT1	VEGFA
C1QB	CD34	CXCL2	FTH1	IGHG4_secreted	ITGB2	NCAM1	STAT3	VMO1
C10orf54	CD36	CXCL3	FUT4	IGHM_membrane	ITK	NCR3	STAT4	VNN2
CASP3	CD37	CXCL5	FYB	IGHM_secreted	JCHAIN	NINJ2	STAT5A	VPREB3
CASP5	CD38	CXCL8	FYN	IGKC	JUN	NKG7	STAT6	VPS28
CBLB	CD40	CXCL9	GAB2	IGLC3	JUNB	NRP1	TARP_	VSIG4
CCL1	CD40LG	CXCL10	GAPDH	IKZF1	KCNE3	NT5E	TBX21	XBP1
CCL2	CD44	CXCL11	GATA3	IKZF2	KDELR1	OAS1	TCF4	XCL1
CCL3	CD48	CXCL13	GHR	IL1B	KIAA0101	PASK	TCF7	YBX3
CCL4	CD52	CXCL16	GIMAP2	IL1R2	KIR2DL1	PAX5	TCL1A	ZAP70
CCL5	CD63	CXCR1	GIMAP5	IL1RL1	KIT	PCNA	TGFB1	ZBED2
CCL13	CD69	CXCR2	GIMAP7	IL1RN	KLRB1	PDCD1	TGFB3	ZBTB16
CCL17	CD70	CXCR3	GLG1	IL2	KLRC1	PDIA4	TGFBI	ZNF683
CCL19	CD72	CXCR4	GNAI2	IL2RA	KLRC3	PDIA6	THBD	

CCL20	CD74	CXCR5	GNLY	IL2RB	KLRC4	PECAM1	THBS1	
CCL22	CD79A	CXCR6	GZMA	IL3	KLRF1	PI3	TIAF1	
CCNB1	CD79B	DEFA3	GZMB	IL3RA	KLRG1	PIK3AP1	TIGIT	
CCND2	CD80	DEFA4	GZMH	IL4	KLRK1	PIK3IP1	TK1	
CCR1	CD86	DOCK8	GZMK	IL4R	LAG3	PMCH	TLR2	
CCR2	CD160	DPP4	GZMM	IL5	LAIR2	POU2AF1	TLR7	

Supplementary Table 6: BD's Seven Bridges Genomics platform output: CITE-seq dataset of 60,957 cells of two healthy donors (D1 and D2) with gene expression and antibody capture measurement.

Sample	Library	Number of Cells	Number of Features
D1	Gene Expression	37,579	431
D1	Antibody Capture	37,579	47
D2	Gene Expression	23,378	431
D2	Antibody Capture	23,378	47
