# Supplementary material for: CRISPR/Cas9 editing of NKG2A improves the efficacy of primary CD33-directed chimeric antigen receptor natural killer cells
Source: Nat Commun. 2024 Sep 30;15:8439. doi: 10.1038/s41467-024-52388-1 (PMC11442982; doi:10.1038/s41467-024-52388-1)
Supplement: Supplementary file 3 — Description of Additional Supplementary Files [file 41467_2024_52388_MOESM3_ESM.pdf]

Description of Additional Supplementary Files

File Name: **Supplementary Movie 1**

Description: Movie using the IncuCyte®S3 microscope platform. Recording of 26.5h incubated GFP^+ OCI-AML2 cells (green) as spontaneous lysis control. Viable GFP^+ OCI-AML2 tumor cells are shown in green based on their GFP expression. Apoptotic cells are labeled in red by Annexin V staining.

File Name: **Supplementary Movie 2**

Description: Movie using the IncuCyte®S3 microscope platform. Recording of 26.5h killing assays of NT-NK cells (unstained) incubated with GFP^+ OCI-AML2 cells (green) in effector to target ratio (E:T) of 0.5:1. Viable GFP^+ OCIAML2 tumor cells appear green due to their GFP expression. Apoptotic tumor cells are labeled in red by Annexin V staining.

File Name: **Supplementary Movie 3**

Description: Movie using the IncuCyte®S3 microscope platform. Recording of 26.5h killing assays of *KLRC1*^{ko}-NK cells (unstained) incubated with GFP^+ OCI-AML2 cells (green) in effector to target ratio (E:T) of 0.5:1. Viable GFP^+ OCI-AML2 tumor appear green due to their GFP expression. Apoptotic tumor cells are labeled in red by Annexin V staining.

File Name: **Supplementary Movie 4**

Description: Movie using the IncuCyte®S3 microscope platform. Recording of 26.5h killing assays of CAR33-NK cells (unstained) incubated with GFP^+ OCI-AML2 cells (green) in effector to target ratio (E:T) of 0.5:1. Viable GFP^+ OCIAML2 tumor cells appear green due to their GFP expression. Apoptotic tumor cells are labeled in red by Annexin V staining.

File Name: **Supplementary Movie 5**

Description: Movie using the IncuCyte®S3 microscope platform. Recording of 26.5h killing assays of CAR33-*KLRC1*^{ko}-NK cells (unstained) incubated with GFP^+ OCI-AML2 cells (green) in effector to target ratio (E:T) of 0.5:1. Viable GFP^+ OCI-AML2 tumor cells appear green due to their GFP expression. Apoptotic tumor cells are labeled in red by Annexin V staining.
