# Supplementary material for: CRISPR/Cas9 editing of NKG2A improves the efficacy of primary CD33-directed chimeric antigen receptor natural killer cells
Source: Nat Commun. 2024 Sep 30;15:8439. doi: 10.1038/s41467-024-52388-1 (PMC11442982; doi:10.1038/s41467-024-52388-1)
Supplement: Supplementary file 9 — Reporting Summary [file 41467_2024_52388_MOESM9_ESM.pdf]

nature portfolio

Corresponding author(s): DAPR - NCOMMS-23-02540B
Last updated by author(s): Jul 26, 2024

Reporting Summary

Nature Portfolio wishes to improve the reproducibility of the work that we publish. This form provides structure for consistency and transparency in reporting. For further information on Nature Portfolio policies, see our Editorial Policies and the Editorial Policy Checklist.

Statistics

For all statistical analyses, confirm that the following items are present in the figure legend, table legend, main text, or Methods section.

n/a	Confirmed	
☐	☒	The exact sample size (*n*) for each experimental group/condition, given as a discrete number and unit of measurement
☐	☒	A statement on whether measurements were taken from distinct samples or whether the same sample was measured repeatedly
☐	☒	The statistical test(s) used AND whether they are one- or two-sided<br>*Only common tests should be described solely by name; describe more complex techniques in the Methods section.*
☒	☐	A description of all covariates tested
☒	☐	A description of any assumptions or corrections, such as tests of normality and adjustment for multiple comparisons
☐	☒	A full description of the statistical parameters including central tendency (e.g. means) or other basic estimates (e.g. regression coefficient) AND variation (e.g. standard deviation) or associated estimates of uncertainty (e.g. confidence intervals)
☐	☒	For null hypothesis testing, the test statistic (e.g. *F*, *t*, *r*) with confidence intervals, effect sizes, degrees of freedom and *P* value noted<br>*Give P values as exact values whenever suitable.*
☒	☐	For Bayesian analysis, information on the choice of priors and Markov chain Monte Carlo settings
☒	☐	For hierarchical and complex designs, identification of the appropriate level for tests and full reporting of outcomes
☒	☐	Estimates of effect sizes (e.g. Cohen's *d*, Pearson's *r*), indicating how they were calculated

Our web collection on statistics for biologists contains articles on many of the points above.

Software and code

Policy information about availability of computer code

Data collection

BD FACSCanto™ (II) /Celesta™ and Cytek Aurora cell analyzer and BDFACSAria™ Fusion II cell sorter were used to collect flow cytometry data and sort cells. In vivo bioluminescence signal was analyzed using an IVIS® Lumina II Multispectral Imaging System. Real-time live-cell imaging was analyzed using a IncuCyte© S3 system. - PCR (on-target amplification) has been performed using the PCR Thermocycler T professional TRIO_ Biometra, Agarose gel electrophersis (MinipowerPack_PS300T_Biometra) and Agarose gel picture acquisition (Fusion FX_Vilber). BM microscopy was performed using a Zeiss Axio Observer Z1. qRT-PCR data was collected using a QuantStudio™ Flex 7 qRT-PCR cycler. Single-cell sequencing data was collected using a BD Rhapsody™ Single-cell analysis system. Sinlge cell libraries were sequenced using a NextSeq™2000 system. BD FACSVerseTM cytometer (BD Biosciences-Pharmingen, USA) was used to perform cytokine bead arrays (CBA).

Data analysis

FlowJo V10, living image V4.5, GraphPad PRISM V6-9, Microsoft Excel/Word/Powerpoint, FCAP Array Software v3.0, IncuCyte Live-cell analysis software, Zeiss Zen Blue, Image J., ICE CRISPR analysis tool by Synthego. For CITE-seq: After sequencing, FASTQ files were generated using BaseSpace Sequence Hub (Illumina, https://basespace.illumina.com). FASTQ Files were uploaded to the SevenBridges platform (Seven Bridges Genomics, https://www.sevenbridges.com) and processed using the BD Rhapsody analysis pipeline (BD Biosciences) with default settings to generate recursive substitution error correction (RSEC)-adjusted molecule count matrices. CITE-seq data analysis was performed on: R version 4.2.2 Patched (2022-11-10 r83330) Platform: x86_64-pc-linux-gnu (64-bit) Running under: Ubuntu 20.04.5 LTS. The R packages/software used: edgeR (Robinson et al., 2010), limma (Ritchie et al., 2015), tidyverse (Wickham et al., 2019), SingleCellExperiment (Amezquita et al., 2020), scran (Lun et al., 2016), scater (McCarthy et al., 2017), igraph (Csardi & Nepusz, 2006), ggplot2 (Wickham, 2016), pheatmap (Kolde, 2019), patchwork (Pedersen, 2022), cowplot (Wilke, 2020), ggrepel (Slowikowski, 2022), ggplotify (Yu, 2021), ggpubr (Kassambara, 2022), magrittr (Bache & Wickham, 2022), scDblFinder (Germain et al., 2021). The version of all the R packages/software used and session info is printed out in the scripts' corresponding html files. All scripts and their html and intermediate files are deposited in this public GitHub repository: https://github.com/AGImkeller/CD33_NK_cells_2022.git

For manuscripts utilizing custom algorithms or software that are central to the research but not yet described in published literature, software must be made available to editors and reviewers. We strongly encourage code deposition in a community repository (e.g. GitHub). See the Nature Portfolio guidelines for submitting code & software for further information.

Data

Policy information about availability of data

All manuscripts must include a data availability statement. This statement should provide the following information, where applicable:

- Accession codes, unique identifiers, or web links for publicly available datasets
- A description of any restrictions on data availability
- For clinical datasets or third party data, please ensure that the statement adheres to our policy

Provide your data availability statement here.

Research involving human participants, their data, or biological material

Policy information about studies with human participants or human data. See also policy information about sex, gender (identity/presentation), and sexual orientation and race, ethnicity and racism.

Reporting on sex and gender

Primary NK cells were isolated from peripheral blood of male donors.

Reporting on race, ethnicity, or other socially relevant groupings

No selection of race, ethnicity or socially relelvant groupings have been done.

Population characteristics

Describe the covariate-relevant population characteristics of the human research participants (e.g. age, genotypic information, past and current diagnosis and treatment categories). If you filled out the behavioural & social sciences study design questions and have nothing to add here, write "See above."

Recruitment

NK cells were isolated from buffy coats of fresh blood from healthy, anonymous donors provided by the German Red Cross Blood Donation (DRK-Blutspendedienst Baden-Württemberg-Hessen, Frankfurt am Main, Germany). Primary AML patient material was obtained from voluntary donors which were treated at the University Hospital Frankfurt.

Ethics oversight

All studies using human material were approved by the local ethical review board (approval no 329/10 and 274/18) and were performed in accordance with the regulations of Helsinki. The use of bone marrow aspirates was approved by the Ethics Committee of University Hospital Frankfurt (approval No. SHN-12-2016, amend 01 2021 and 02 2022) and the study alliance leukemia (SAL, approval No. EK98032010). All mice experiments were approved by the Regierungspräsidium Darmstadt, Germany (FK1123).

Note that full information on the approval of the study protocol must also be provided in the manuscript.

Field-specific reporting

Please select the one below that is the best fit for your research. If you are not sure, read the appropriate sections before making your selection.

☒ Life sciences ☐ Behavioural & social sciences ☐ Ecological, evolutionary & environmental sciences

For a reference copy of the document with all sections, see nature.com/documents/nr-reporting-summary-flat.pdf

Life sciences study design

All studies must disclose on these points even when the disclosure is negative.

Sample size

No statistical methods were used to predetermine sample size. Sample sizes were estimated based on preliminary results/experiments. For in vivo experiments the treatment effects were distinct and matched with preliminary results. Thus a sample size of n=4 mice per group was sufficient to show the treatment advantage and reduce the number of used animals.

Data exclusions	Animals which did not show tumor cell engraftment post tumor cell injection were excluded.
Replication	All important in vitro experiments were repeated at least 3-5 times. For some experiments it was beneficial to display less than three experiments, due to CAR-NK cell donor dependent heterogeneities. Two animal experiments were performed independently and are shown.
Randomization	Allocation was random by buffy coats of fresh blood from healthy, anonymous donors provided by the German Red Cross Blood Donation (DRK-Blutspendedienst Baden-Württemberg-Hessen, Frankfurt am Main, Germany) and no selection process was done.<br>For in vivo experiments all mice were imaged routinely pre and post (CAR-)NK cell infusions. Mice included in the same experiment always had the same sex and approximately the same age (10-16 weeks old at the start of experiment). To achieve homogenous tumor cell engraftments in each group, mice were randomized by distributing them equally to each group depending on the total flux analysis at d2 post tumor cell injection.
Blinding	No blinding during the experiment was possible in this study, due to limited numbers of executing experimenters. However, all the obtained data are objective measurements measured by technical instruments.

Reporting for specific materials, systems and methods

We require information from authors about some types of materials, experimental systems and methods used in many studies. Here, indicate whether each material, system or method listed is relevant to your study. If you are not sure if a list item applies to your research, read the appropriate section before selecting a response.

Materials & experimental systems

n/a	Involved in the study	
☐	☒	Antibodies
☐	☒	Eukaryotic cell lines
☒	☐	Palaeontology and archaeology
☐	☒	Animals and other organisms
☐	☒	Clinical data
☒	☐	Dual use research of concern
☒	☐	Plants

Methods

n/a	Involved in the study	
☒	☐	ChIP-seq
☐	☒	Flow cytometry
☒	☐	MRI-based neuroimaging

Antibodies

Antibodies used	All used antibodies are described specifcly in the Supplementary Tables 5 and the "Method" section of the manuscript.
Validation	All used antibodies were deployed according to manufacturer's instructions or were titrated to achieve the best resolution with minimal unspecific binding and minimal antibody consumptions. The best titration was determined by comparing the positive controls (e.g. PBMC or immune cells) with the negative controls (such as cells stained with isotype or FMO controls). All used antibodies are validated for their use in either flow cytometry, western blot or CITE-seq analysis. All used antibodies are commercially available.

Eukaryotic cell lines

Policy information about cell lines and Sex and Gender in Research

Cell line source(s)	OCI-AML2 and HEK293T cell lines were obtained from DSMZ-German Collection of Microorganisms and Cell Cultures.
Authentication	DSMZ use smorphology, PCR and STR assays to authenticate cell lines. Addtiononally, morphology, viability and experiment-related antigen expression was confirmed routinely by flow cytometry.
Mycoplasma contamination	All cell lines were regularly tested for mycoplasma contamination and found to be negative.
Commonly misidentified lines (See ICLAC register)	No commonly misidentified cell lines were used in this study.

Animals and other research organisms

Policy information about studies involving animals; ARRIVE guidelines recommended for reporting animal research, and Sex and Gender in Research

Laboratory animals	10-16 weeks old NOD.Cg-Prkdcscid Il2rgtm1Wjl Tg(CMV-IL3,CSF2,KITLG)1Eav/MloySzJ (NSG-SGM3) mice obtained from The Jackson Laboratory were used.
Wild animals	No field-collected samples were applied in this study.

Reporting on sex	Mice included in one experiment where chosen from the same sex to exclude sex-dependent differences. It is not possible to conclude from this study whether our findings apply only to one sex, due to sample size and available animals. However, taken together all animal experiments included in this study as well as previous experiments with CAR33-Nk cells, we can assume that CAR33-NK and CAR33-KLRC1ko-NK cells are effective in mice of both sex.
Field-collected samples	No field-collected samples were applied in this study.
Ethics oversight	All animal experiments were approved by the Regierungspräsidium Darmstadt, Germany.

Note that full information on the approval of the study protocol must also be provided in the manuscript.

Clinical data

Policy information about clinical studies

All manuscripts should comply with the ICMJE guidelines for publication of clinical research and a completed CONSORT checklist must be included with all submissions.

Clinical trial registration	*Provide the trial registration number from ClinicalTrials.gov or an equivalent agency.*
Study protocol	*Note where the full trial protocol can be accessed OR if not available, explain why.*
Data collection	*Describe the settings and locales of data collection, noting the time periods of recruitment and data collection.*
Outcomes	*Describe how you pre-defined primary and secondary outcome measures and how you assessed these measures.*

Plants

Seed stocks	*Report on the source of all seed stocks or other plant material used. If applicable, state the seed stock centre and catalogue number. If plant specimens were collected from the field, describe the collection location, date and sampling procedures.*
Novel plant genotypes	*Describe the methods by which all novel plant genotypes were produced. This includes those generated by transgenic approaches, gene editing, chemical/radiation-based mutagenesis and hybridization. For transgenic lines, describe the transformation method, the number of independent lines analyzed and the generation upon which experiments were performed. For gene-edited lines, describe the editor used, the endogenous sequence targeted for editing, the targeting guide RNA sequence (if applicable) and how the editor was applied.*
Authentication	*Describe any authentication procedures for each seed stock used or novel genotype generated. Describe any experiments used to assess the effect of a mutation and, where applicable, how potential secondary effects (e.g. second site T-DNA insertions, mosiacism, off-target gene editing) were examined.*

Flow Cytometry

Plots

Confirm that:

☒ The axis labels state the marker and fluorochrome used (e.g. CD4-FITC).

☒ The axis scales are clearly visible. Include numbers along axes only for bottom left plot of group (a 'group' is an analysis of identical markers).

☒ All plots are contour plots with outliers or pseudocolor plots.

☒ A numerical value for number of cells or percentage (with statistics) is provided.

Methodology

Sample preparation	NK cell isolation is described in the "Methods" section. Cell staining was performed in Brilliant Stain Buffer (BD Biosciences). Fc-receptor blockade was performed by addition of unspecific hIgG (Kiovig or Intratect®). CAR expression analysis is described in the 'Methods' section. To distinguish AML cells from NK cells, AML cells were stained with CellTrace™ CFSE - Cell Proliferation Kit (ThermoFisher) prior to co-cultivation. Preparation of BM cells and splenocytes from mice is described in the 'Methods' section. Prior to flow cytometry analysis cells were washed with PBS and taken up in FACS buffer (Cell Wash (BD Biosciences) + 0.5% BSA + 0.001% NaN3). Cell viability was assessed by addition of 7-AAD (BD Biosciences) or DAPI (AppliChem).
Instrument	For data collection BD FACSCanto™, BD FACSCelesta™ cell analyzer and BDFACSAria™ Fusion II cell sorter were used.
Software	FlowJo (10.3) software was used to analyze flow data.
Cell population abundance	Post-sort populations achieved over 95% purity, which was analyzed by flow cytometry. Functional assays with NK were only performed with >90% purity of the NK cell product.
Gating strategy	Relevant gating strategies are displayed in Figure 1a and Supplementary Figure 1a, S4e, S5b. Positive gates were either

Gating strategy	identified whene two clearly distinguishable populations were present and/or set on negative controls (e.g. isotype or FMO controls).

☒ Tick this box to confirm that a figure exemplifying the gating strategy is provided in the Supplementary Information.
